# Olink proteomics identifies serum protein biomarkers and an age-stratified diagnostic model for diabetes in Chinese patients

**DOI:** 10.3389/fendo.2026.1900153

**Published:** 2026-08-26

**Authors:** Yan Yang, Shiyu Liu, Chunguang Xie, Danping Zhu

**Affiliations:** 1 Chongqing Traditional Chinese Medicine Hospital, Chongqing, China; 2 Hospital of Chengdu University of Traditional Chinese Medicine, Chengdu, China; 3 Mianyang Traditional Chinese Medicine Hospital, Mianyang, China

**Keywords:** type 2 diabetes meliitus (T2DM), biomarkers, proteomics, age stratification, biological aging

## Abstract

**Background:**

Aging is a critical risk factor for the progression and complications of type 2 diabetes mellitus (T2DM). However, routine clinical indicators fail to accurately quantify biological senescence burden in T2DM patients. This study aimed to screen plasma protein signatures associated with metabolic senescence in T2DM using Olink targeted proteomics and to construct a novel biological aging evaluation model for diabetic populations.

**Methods:**

A total of 21 healthy controls and 66 T2DM patients were enrolled. Plasma protein profiles were detected via Olink proteomics. Differentially expressed proteins (DEPs) were identified and subjected to GO and KEGG functional enrichment analyses. Random forest and LASSO regression analyses were applied to screen core T2DM-related protein molecules. T2DM patients were further stratified by a 55-year cutoff, a well-recognized critical threshold for metabolic senescence. After adjustment for sex, BMI, glycated hemoglobin (HbA1C), and hypoglycemic medication use, age-independent senescence-related proteins were identified to establish a multi-protein predictive model. A 1000-time Bootstrap resampling procedure was performed for internal validation to evaluate model discrimination and stability.

**Results:**

A total of 84 DEPs (P < 0.05) were identified between T2DM patients and healthy controls, mainly enriched in cytokine–cytokine receptor interaction, lipid metabolism, and atherosclerosis pathways. Machine learning screened six core proteins with high discriminatory value for T2DM, including MERTK, BOC, TNFRSF10A, CCL3, AGRP, and PD-L2. Within the T2DM cohort, nine age-dependent plasma proteins were independently identified (FDR < 0.05), among which VSIG2, LPL, S100A11, and CST5 exhibited the most robust correlations (FDR < 0.01). A three-protein model comprising VSIG2, S100A11, and S100A5 achieved favorable discriminative performance (AUC = 0.874), which was superior to conventional clinical indicators including BMI (AUC = 0.572) and HbA1c (AUC = 0.593). Bootstrap validation confirmed reliable model stability with a correction optimism of −0.024, a bias-corrected AUC of 0.900, and an overfitting degree of −2.65%. Functional analyses indicated that candidate proteins were primarily involved in immune homeostasis, lipid remodeling, inflammatory responses, and calcium signaling.

**Conclusion:**

This study identified novel T2DM-associated plasma biomarkers and established a robust three-protein signature (VSIG2, S100A11, S100A5) for age stratification and biological senescence evaluation in T2DM. The proposed model compensates for the limitations of routine clinical indices, providing a promising non-invasive serological tool for precise risk stratification and individualized intervention in elderly diabetic patients.

## 1 Introduction

The global prevalence of diabetes remains persistently high. The number of individuals living with diabetes was estimated at 537 million in 2021, with projections of 643 million by 2030 and 783 million by 2045 (1). Over the past three decades, diabetes prevalence has increased significantly in China (2). According to the Chinese National Center for Chronic and Non-communicable Disease Prevention and Control, the prevalence of diabetes in China was 11.9% in 2018–2019, and 12.4% based on the 2010 ADA criteria (3). Overweight, obesity, lifestyle changes, and environmental pollution are closely associated with the rising diabetes incidence in China (2).

Long-term hyperglycemia and insulin resistance (IR) lead to dysregulated glucose and lipid metabolism, predisposing patients to various microvascular and macrovascular complications. Approximately one-third of diabetic patients develop retinopathy (4). Compared with healthy individuals, patients with T2DM exhibit a 2-fold higher risk of diabetic kidney disease, a 2–3-fold elevated risk of cardiovascular disease, and a 5-fold greater risk of diabetic foot ulcers (5–7). These complications impose a heavy socioeconomic burden; diabetes accounts for 12% of global healthcare expenditure, exceeding $96 billion annually (8). Accurate risk stratification and timely intervention are therefore critical to reducing medical costs and improving patient prognosis.

Current indicators for T2DM assessment, including fasting plasma glucose (FPG), glycated hemoglobin (HbA1c), and the oral glucose tolerance test (OGTT), have notable inherent limitations. FPG requires prolonged fasting and suffers from substantial biological and circadian variability, reflecting only a snapshot of glycemic status at a single time point (9). While HbA1c is convenient and stable, the diagnostic cutoff of ≥6.5% fails to detect a considerable proportion of early T2DM cases identified by combined FPG and OGTT (10). Furthermore, HbA1c correlates weakly with dynamic IR and insulin secretory capacity (11). As a result, more than half of patients remain undiagnosed until severe complications develop (12). Even among confirmed diabetic individuals, routine metabolic markers cannot adequately evaluate biological aging status. Novel circulating biomarkers are urgently needed to support refined risk stratification and personalized management.

Recent advances in proteomics offer a powerful platform for biomarker discovery and mechanistic exploration. The Olink targeted proteomics platform enables high-throughput, high-sensitivity quantification of circulating cardiovascular and metabolic proteins, rendering it suitable for biomarker screening in metabolic diseases. In diabetes research, Olink proteomics has been successfully applied to predict diabetes-related cardiovascular complications and evaluate therapeutic responses to metformin (13–15).

Aging constitutes a major risk factor for the development of T2DM (16). Cellular senescence acts as a mechanistically distinct driver of IR and β-cell dysfunction (17). Senescent cells accumulate in glucose homeostatic organs including adipose tissue, liver, skeletal muscle and pancreatic islets. These cells continuously secrete pro-inflammatory factors and matrix remodeling mediators, collectively termed the senescence-associated secretory phenotype (SASP) (16). The SASP comprises a complex mixture encompassing pro-inflammatory cytokines (IL-6, IL-1β, TNF-α), chemokines (MCP-1), proteases (MMP-9, MMP-12), profibrotic mediators (PAI-1), and damage-associated molecular patterns such as HMGB1 (18). SASP disrupts insulin signaling, promotes immune cell infiltration, and accelerates pancreatic β-cell failure (17). Furthermore, glucotoxicity, advanced glycation end product (AGE) toxicity, immuno-inflammaging and protein amyloidosis are additional drivers that accelerate senescence throughout T2DM progression (19). Notably, the degree of senescence burden varies markedly among T2DM individuals with identical chronological age. Conventional clinical indicators cannot adequately capture such heterogeneity in biological aging. The age of 55 years represents a critical inflection point for metabolic senescence. This cutoff has been widely adopted in studies investigating aging-related metabolic disorders, making it a rational threshold for subgroup stratification among T2DM cohorts.

In the present study, we first performed proteomic comparison between healthy controls and T2DM patients to characterize diabetes-related protein expression patterns. More importantly, we further stratified T2DM patients using the 55-year cutoff and systematically screened age-dependent differential proteins specifically within the diabetic population. We identified a panel of age-related biomarkers independently associated with diabetes-associated aging, which participate in immune regulation, lipid metabolism and calcium signaling pathways. Based on these core markers, we constructed and thoroughly validated a parsimonious three-protein model for age stratification among T2DM patients. This novel protein signature exhibits superior discriminative ability compared with conventional clinical indices, providing a minimally invasive and reliable tool for evaluating biological aging and achieving precision risk stratification. Our findings deepen mechanistic insights into diabetes-associated aging and offer potential targets for anti-senescence and individualized diabetic intervention.

## 2 Materials and methods

### 2.1 Study subjects and ethical approval

This study was a single-center, observational, case-control study. It was approved by the Ethics Committee of the Affiliated Hospital of Chengdu University of Traditional Chinese Medicine (Sichuan Provincial Hospital of Traditional Chinese Medicine) (approval No. 2023-KL-130). All participants provided written informed consent.

### 2.2 Inclusion and exclusion criteria

#### 2.2.1 Inclusion criteria

##### 2.2.1.1 Type 2 diabetes

Inclusion criteria were as follows: (1) diagnosis of type 2 diabetes according to the 2022 American Diabetes Association (ADA) Standards of Medical Care in Diabetes, meeting any of the following criteria: fasting plasma glucose (FPG) ≥ 126 mg/dL (7.0 mmol/L); 2-hour plasma glucose ≥ 200 mg/dL (11.1 mmol/L) during an oral glucose tolerance test; glycated hemoglobin (HbA1c) ≥ 6.5% (48 mmol/mol); or, in patients with typical hyperglycemic symptoms or a hyperglycemic crisis, a random plasma glucose ≥ 200 mg/dL (11.1 mmol/L). In the absence of unequivocal hyperglycemia, two abnormal test results from the same or two separate samples were required; (2) age between 18 and 65 years; (3) clinical diagnosis of type 2 diabetes confirmed by medical records; and (4) willingness to participate and ability to provide written informed consent.

##### 2.2.1.2 Healthy volunteers

① Age between18 to 65 years old; ② Willing to participate and able to provide written informed consent.

#### 2.2.2 Exclusion criteria

① Presence of acute complications of T2DM (diabetic ketoacidosis, hyperosmolar non-ketotic diabetic coma, lactic acidosis, etc.); ② Severe liver or kidney dysfunction (ALT/AST > 2.5 × upper limit of normal, or Cr > upper limit of normal, or eGFR ≤ 60 μmol/L); ③ Pregnancy or lactation; ④ Presence of foot gangrene, ulcers, or complete occlusion of lower extremity vessels; ⑤ Mental disorders that prevented cooperation with examinations; ⑥ Previous coronary angiography confirming coronary heart disease, or electrocardiogram/clinical manifestations suggestive of coronary heart disease, or a previous definite cerebrovascular accident; ⑦ Long-term anticoagulant therapy; ⑧ Malignant tumors, systemic infectious diseases, immune system diseases, severe psychosomatic diseases, or a life expectancy ≤ 2 years.

### 2.3 Sample collection and storage

Fasting venous blood (5–6 mL) was collected from all subjects using EDTA anticoagulant tubes. After standing for 30 minutes, the blood was centrifuged at 2000 × g for 10 minutes. Plasma was separated in a sterile operating bench, aliquoted into 2 mL cryotubes, and stored at −80 °C for subsequent proteomic analysis.

### 2.4 Olink proteomics

#### 2.4.1 Technical principle

The Olink Target 96 Cardiovascular II panel was used for detection. This panel measures 92 target proteins and 4 internal control proteins. Based on the patented Proximity Extension Assay (PEA) technology, pairs of antibodies conjugated with specific DNA oligonucleotide barcodes were used to specifically detect target proteins.

#### 2.4.2 Experimental procedure

① After the antibody pairs recognized and bound to the target protein, the complementary DNA barcode tails hybridized and were extended by DNA polymerase; ② The newly synthesized DNA barcodes were pre-amplified by PCR, and their types and quantities were detected by qPCR or next-generation sequencing (NGS), enabling specific quantification of the target proteins; ③ Ct values were normalized and standardized using a set of internal controls and external quality control data; ④ Raw data were log2-transformed and presented as Normalized Protein Expression (NPX) values (20).

#### 2.4.3 Quality control

Four internal controls were added to each sample to monitor analytical performance and individual sample quality; ① A normalized protein expression (NPX) value < 0.2 was used as the criterion to assess the standard deviation of the internal controls for each plate; ② A deviation of the control value from the median of < 0.3 NPX per sample was used as the criterion for individual sample quality control; ③ The coefficient of variation (CV) was calculated for each sample; samples with a CV > 20% were considered to have failed quality control.

#### 2.4.4 Data output

The Olink^®^ Target 96 Cardiovascular II panel was used to simultaneously measure 92 cardiovascular disease-related proteins. Quality control and normalization were performed using Olink NPX Manager software. Protein expression levels are presented as NPX (an arbitrary unit on a log2 scale, where higher values indicate higher expression abundance). All samples were analyzed in a single batch to eliminate batch effects (13).

### 2.5 Protein screening strategy for T2DM

#### 2.5.1 Quality control

To assess the quality of the proteomics data, systematic quality control was performed prior to formal analysis. Principal component analysis (PCA) was applied to evaluate the overall distribution of samples and the separation between groups. Sample correlation heatmap combined with hierarchical clustering analysis was used to verify within-group consistency and between-group differences. Orthogonal partial least squares discriminant analysis (OPLS-DA) with 200 permutation tests was employed to determine whether the molecular differences between the two groups were statistically significant. Within-group coefficient of variation (CV) distributions were compared to assess technical reproducibility. All quality control analyses were conducted using R 4.3.1.

#### 2.5.2 Differential protein screening

Differential expression analysis was performed using the limma package. Proteins with raw P < 0.05 were considered candidate DEPs. To control for false positives due to multiple testing, the Benjamini-Hochberg false discovery rate (FDR) method wasapplied, and FDR < 0.05 was used as the final significance threshold for identifying robust DEPs.

#### 2.5.3 Enrichment analysis

GO functional enrichment analysis and KEGG pathway enrichment analysis were performed, and bar plots were generated to elucidate the cellular functions and downstream signaling pathways involving the differential proteins. When fewer than 10 differential proteins were identified, the online tool MetaboAnalyst (https://www.metaboanalyst.ca/home.xhtml) was used for enrichment analysis.

#### 2.5.4 Random forest for screening key differential proteins

To further evaluate the discriminatory capacity of the 16 candidate differentially expressed proteins and identify core features, we employed the random forest (RF) machine learning algorithm for feature importance ranking. RF is an ensemble learning method based on bootstrap aggregating (bagging), which constructs multiple independent decision trees through bootstrap sampling and combines their classification results to achieve stable classification with low overfitting. All RF analyses were performed using the randomForest package in R, with the number of trees set to 500 (ntree = 500) and a fixed random seed (set.seed = 123) to ensure reproducibility. The Mean Decrease Gini (MDG) index was used to quantify the classification importance of each protein; a higher MDG value indicates stronger discriminatory power.

#### 2.5.5 LASSO regression to improve differential protein selection efficiency

LASSO (Least Absolute Shrinkage and Selection Operator) regression, an L1-regularized linear method, shrinks coefficients of irrelevant or redundant features to zero by adding a penalty term (L1 norm) to the loss function, thereby achieving variable selection and model complexity control. The implementation steps are as follows: first, all independent variables are Z-score standardized to eliminate scale differences; then, 10-fold cross-validation is used to search the logarithmic space of λ for the optimal penalty parameter (λ.min) that minimizes the cross-validation error; a coefficient path is plotted to visualize the dynamic change of each variable’s coefficient with λ; finally, features with non-zero coefficients at λ.min are retained as the selected key variables. This approach effectively handles multicollinearity in high-dimensional small-sample data, enables automatic feature selection, and enhances model interpretability. Combining LASSO regression with random forest can significantly improve the reliability of candidate biomarker screening.

### 2.6 Protein screening strategy for age-stratified T2DM analysis

#### 2.6.1 Differential protein screening

Differentially expressed proteins were identified using the following criteria: ① Wilcoxon rank-sum test, with P < 0.05 as the initial screening threshold; ② Benjamini-Hochberg false discovery rate (FDR) correction, with FDR < 0.05 considered statistically significant; ③ |log_2_FC| > 0.585 (fold change > 1.5); and ④ variable importance in projection (VIP) > 1.0 from the OPLS-DA model. Proteins meeting all four criteria were designated as differentially expressed proteins.

#### 2.6.2 Multivariable adjustment models

To exclude potential confounding effects, four stepwise-adjusted linear regression models were constructed to evaluate the association between age group (<55 vs ≥55 years) and protein expression: Model 1 was unadjusted; Model 2 adjusted for sex, BMI, and HbA1c; Model 3 further adjusted for medication use (yes/no); and Model 4 further adjusted for metformin and insulin use. β coefficients represent changes in protein expression in the ≥55 years group relative to the <55 years group. Multiple testing was corrected using the Benjamini-Hochberg method, with FDR < 0.05 considered statistically significant.

#### 2.6.3 Multi-protein panel construction and optimization

Based on the four core proteins with FDR < 0.01 after multivariable adjustment (VSIG2, LPL, S100A11, and CST5), a multi-protein logistic regression model was developed. Stepwise regression with Akaike information criterion (AIC) minimization was employed to select the optimal protein combination. Model discrimination was evaluated using the area under the receiver operating characteristic curve (AUC), and the optimal cutoff value was determined by the Youden index. To further optimize the model, S100A5 was introduced to replace redundant proteins, yielding the final 3-protein panel (VSIG2 + S100A11 + S100A5).

#### 2.6.4 Model validation

Bootstrap internal validation with 1000 resamples was performed to assess model stability. Overfitting risk was evaluated by calculating the optimism (difference between the original AUC and the Bootstrap mean AUC). The overfitting index was calculated as (original AUC − corrected AUC)/original AUC × 100%, with values < 5% indicating no significant overfitting. Model calibration was assessed using the Hosmer-Lemeshow goodness-of-fit test.

### 2.7 Data statistics

Statistical analyses were performed using R software (version 4.3.1). Continuous variables were compared using the independent samples t-test or Mann–Whitney U test, as appropriate, and categorical variables were compared using the Chi-square test. Differentially expressed proteins were identified using the Wilcoxon rank-sum test, with FDR correction (Benjamini–Hochberg) for multiple testing. Multivariable linear regression models with stepwise adjustment were used to control for confounding factors. Machine learning analyses were performed using the randomForest and glmnet packages. Model performance was evaluated using AUC, and internal validation was conducted using Bootstrap (1000 resamples) and repeated 10-fold cross-validation (100 repetitions). All statistical tests were two-sided, and P < 0.05 was considered statistically significant.

## 3 Results

### 3.1 Differentially expressed proteins between healthy volunteers and T2DM patients

#### 3.1.1 Baseline clinical characteristics of T2DM vs healthy volunteers

A total of 87 participants were enrolled, including 21 healthy controls and 66 patients with T2DM. Baseline clinical characteristics are summarized in **Table 1**. The two groups were well-balanced in terms of age and sex (both P > 0.05), confirming their comparability. As expected, the T2DM group showed significantly elevated fasting plasma glucose and glycated hemoglobin (HbA1c) levels (both P < 0.001), validating the accuracy of group assignment. Anthropometric evaluation revealed that the T2DM group had significantly greater body weight and waist circumference (both P < 0.05), indicating central obesity as a key metabolic feature of this diabetic population. No significant difference was observed in BMI between groups.

**Table 1:** Baseline clinical characteristics of the study population.

Variable	Normal (n = 21)	Diabetes (n = 66)	P value
Age, median [IQR]	50.48 ± 12.56	50.88 ± 11.03	0.831
Male, n (%)	8 (38.1)	43 (65.2)	0.053
BMI, kg/m²	23.81 ± 2.83	24.98 ± 3.36	0.244
Waist circumference, cm	83.95 ± 11.02	91.22 ± 9.80	< 0.05
Hip circumference, cm	92.67 ± 18.43	99.09 ± 7.44	0.102
FPG, mmol/L	5.35 ± 0.64	9.44 ± 3.39	< 0.001
HbA1c, %	5.10 ± 0.49	9.01 ± 2.52	< 0.001

#### 3.1.2 Olink targeted proteomics quality control

**Figure 1:**
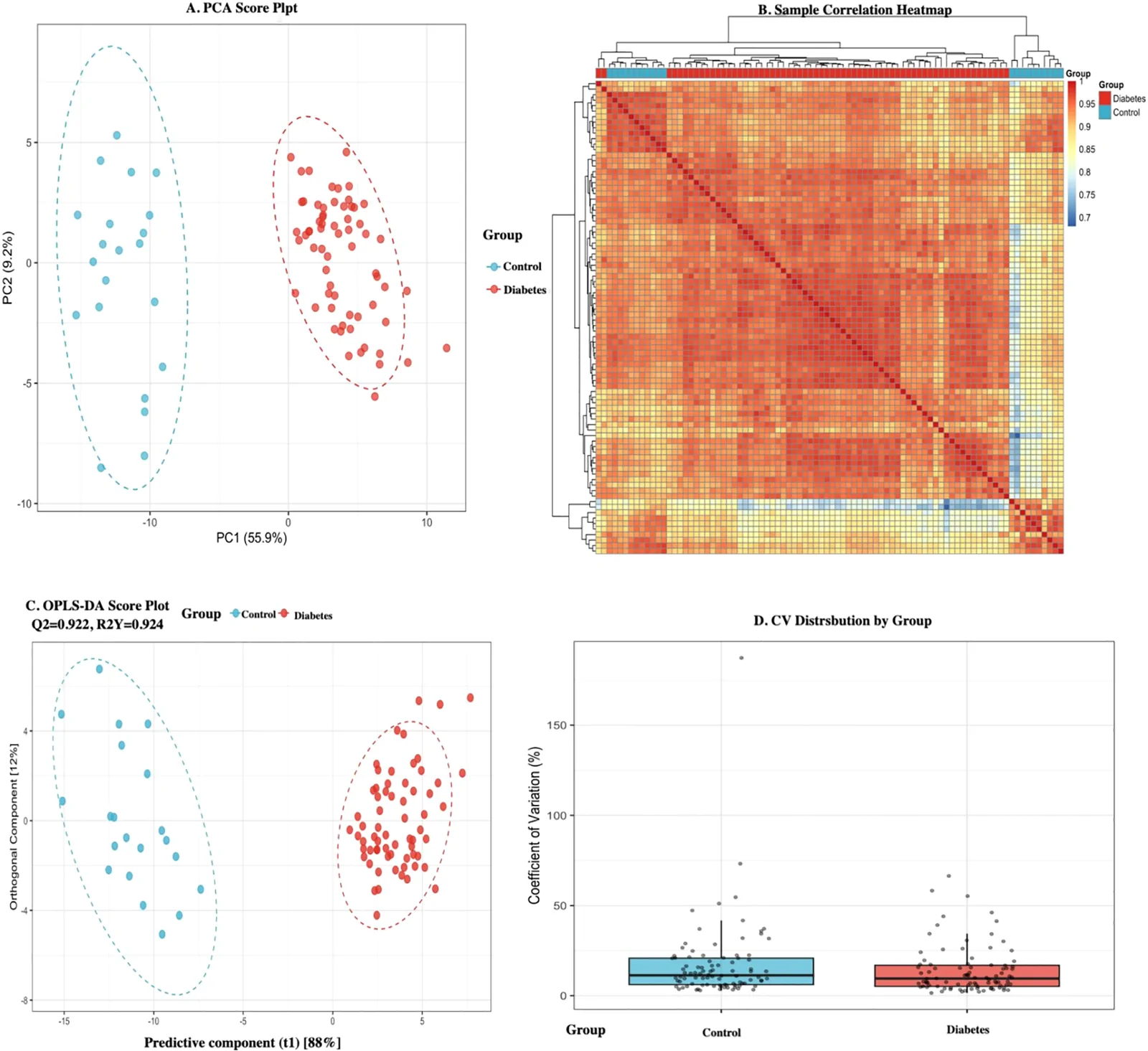
Quality control and group separation of control and diabetes samples. **(A)** PCA score plot; **(B)** sample correlation heatmap; **(C)** OPLS-DA score plot; **(D)** CV distribution boxplot. The blue color represents the control group, and the red color represents the diabetes group.

Prior to formal data analysis, quality control of the proteomics data was performed. **Figure 1A** shows the principal component analysis (PCA) score plot, in which PC1 and PC2 explained 55.9% and 9.2% of the total variance, respectively; the control (blue) and diabetes (red) samples were clearly separated in the PCA space, with good within-group clustering and no obvious outliers, indicating a substantial difference in global molecular profiles between the two groups. **Figure 1B** presents the sample correlation heatmap, revealing a clear “high within-group correlation, low between-group correlation” pattern, and clustering analysis divided the samples into two distinct clusters, further validating the group differences. **Figure 1C** displays the orthogonal partial least squares discriminant analysis (OPLS-DA) score plot, where the model parameters R²Y = 0.924 and Q² = 0.922 (200 permutations, P < 0.001 for both R² and Q²) indicate excellent goodness-of-fit and predictive ability. The two groups were almost completely separated on the predictive component axis, with non-overlapping 95% confidence ellipses, confirming stable and model-detectable differences between the groups. **Figure 1D** shows the boxplot of within-group coefficient of variation (CV) distribution; the CV values for both control and diabetes groups were low, and the distributions did not differ significantly between groups, demonstrating good experimental reproducibility and minimal influence of technical variation on subsequent analyses. Collectively, these results confirm the high quality of the sample data and the existence of stable, significant molecular differences between the control and diabetes groups, providing a solid foundation for subsequent differential protein screening and functional enrichment analysis.

#### 3.1.3 Serum differential protein screening

**Figure 2:**
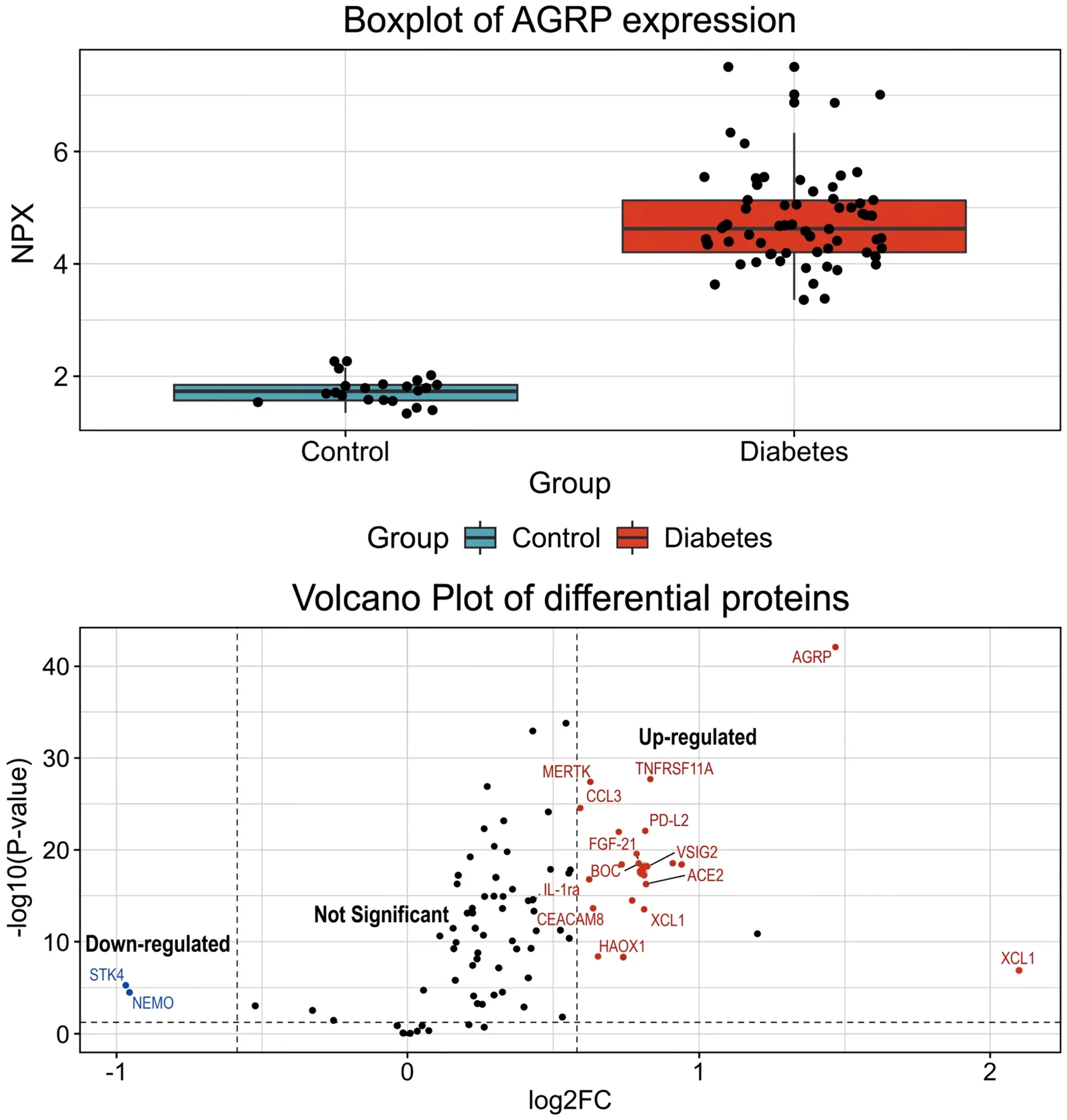
Volcano plot of differential proteins and AGRP expression. The right panel shows the volcano plot of the 16 differential proteins, where red dots represent significantly up-regulated proteins, blue dots represent significantly down-regulated proteins, and gray dots represent non-significant proteins.

As shown in **Figure 2**, a total of 84 proteins showed differential expression with raw P < 0.05. To control for false positives due to multiple testing, we applied Benjamini-Hochberg false discovery rate (FDR) correction. Using the stringent triple-filtering strategy of |log_2_FC| > 0.585 (fold change > 1.5), variable importance in projection (VIP) > 1.0, and FDR < 0.05, 16 proteins were identified as significantly differentially expressed proteins (DEPs). Among these, 14 were upregulated in the diabetes group (AGRP, ACE2, VSIG2, TNFRSF11A, PD-L2, XCL1, FGF-21, BOC, TNFRSF10A, HAOX1, CEACAM8, MERTK, IL-1ra, and CCL3), while 2 were downregulated (NEMO and STK4).

#### 3.1.4 Enrichment analysis of differential proteins

**Figure 3:**
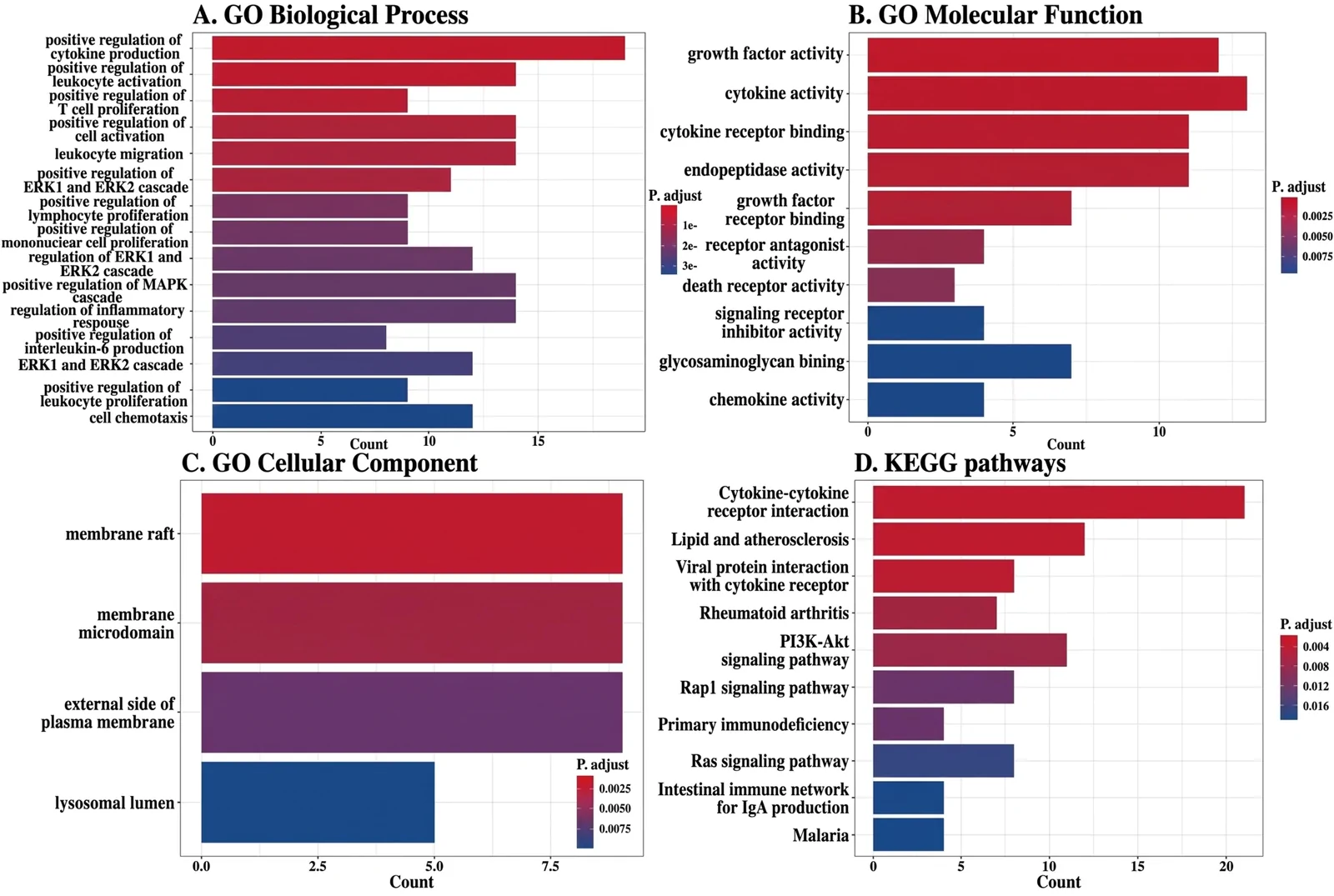
GO and KEGG enrichment analyses of 84 differentially expressed proteins. **(A–C)** GO enrichment showed that these proteins were mainly involved in immune and inflammatory responses at the biological process level, exerted cytokine- and growth factor-related functions at the molecular function level, and were largely localized to membrane rafts and membrane microdomains in cellular components. **(D)** KEGG analysis identified cytokine-cytokine receptor interaction and lipid and atherosclerosis as the major enriched pathways.

Enrichment analysis of the 84 differential proteins was performed to obtain comprehensive biological insights. GO biological process (BP) terms were mainly enriched in immune and inflammatory response regulation, including positive regulation of cytokine production, leukocyte activation/proliferation/migration, T cell proliferation, cell activation, and regulation of the ERK1/ERK2 cascade, suggesting that the differential proteins play key roles in inflammation activation and immune cell recruitment and activation. GO molecular function (MF) terms were predominantly related to cytokine and growth factor activities, including cytokine activity, growth factor activity, cytokine receptor binding, and endopeptidase activity, indicating that the differential proteins mediate signaling and effector functions mainly through regulating cytokine/growth factor activity and binding. GO cellular component (CC) terms were enriched in membrane-associated structures, such as membrane raft and membrane microdomain, implying that the differential proteins are primarily localized to specialized membrane microdomains involved in signal complex assembly and transduction (**Figures 3A–C**). KEGG pathway enrichment analysis revealed that the differential proteins of diabetes identified in this study primarily mediate chronic inflammation through cytokine-receptor interactions, and coordinately regulate lipid metabolism, the PI3K-Akt insulin signaling pathway, and systemic immune homeostasis. Chronic inflammation, lipid metabolism disorders, and insulin signaling abnormalities collectively constitute the key pathological features of diabetes (**Figure 3D**).

#### 3.1.5 Ranking of the key differentially expressed proteins

This study employed the random forest (RF) machine learning algorithm to rank the feature importance and evaluate the disease-discriminatory ability of the 16 candidate differential proteins. RF is an ensemble learning method based on multiple decision trees. By using bootstrap sampling with replacement, it constructs many independent decision trees and combines their classification results to achieve stable classification with low overfitting, thereby objectively assessing the contribution of each feature to group discrimination.

**Figure 4:**
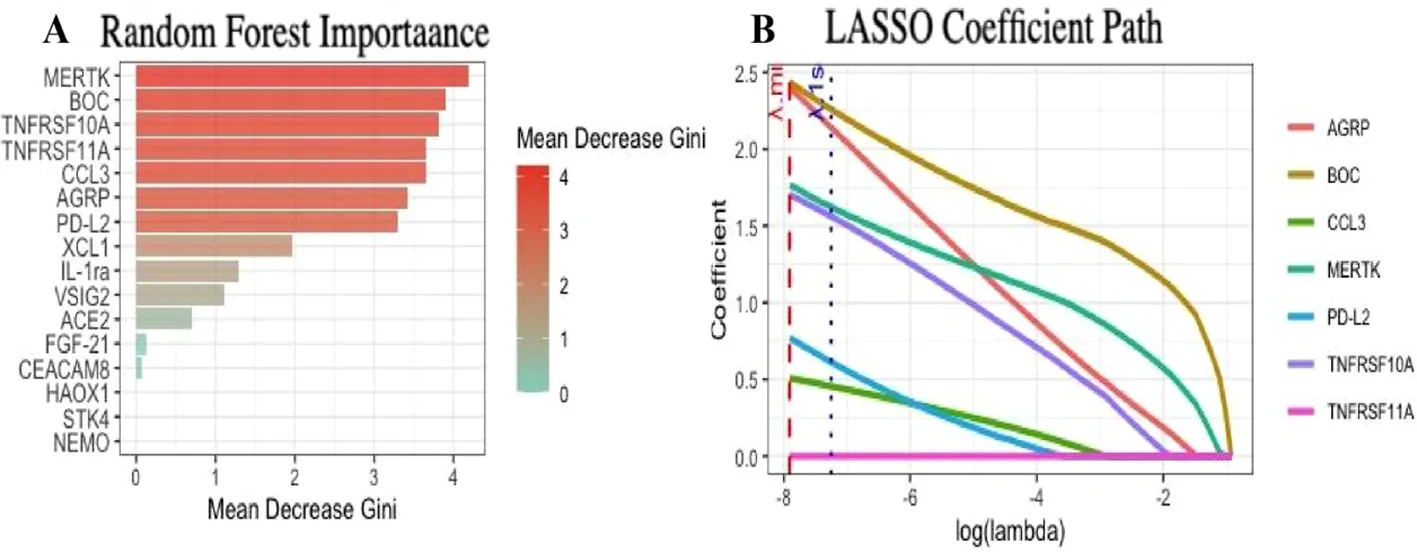
Integration of random forest and LASSO for core diagnostic protein selection. **(A)** Random forest variable importance (Mean Decrease Gini) for 16 differential proteins **(B)** LASSO coefficient paths for the seven candidates.

All analyses were performed using the randomForest package in R (version 4.3.1). The number of trees was set to 500 (ntree = 500), and the number of variables randomly sampled as candidates at each split was set to 4 (mtry = 4), corresponding to the square root of the total number of candidate features (√16 = 4) as per the default recommendation for classification tasks. A fixed random seed (set.seed = 123) was used to ensure reproducibility. Variable importance was evaluated using the Mean Decrease Gini (MDG) index, where a higher MDG value indicates stronger discriminatory power for distinguishing between the two groups and greater biological discriminant value. The results showed that MERTK, BOC, TNFRSF10A, TNFRSF11A, CCL3, AGRP, and PD-L2 all had MDG values > 3, identifying them as highly important proteins in discriminating T2DM from healthy controls (**Figure 4A**).

To further refine this candidate set and construct an interpretable prediction model, we performed feature selection using LASSO (Least Absolute Shrinkage and Selection Operator) logistic regression. LASSO introduces an L1 regularization penalty (alpha = 1) that compresses the regression coefficients of uninformative or redundant features to zero, thereby enabling automatic variable selection and model dimensionality reduction. The analysis was conducted using the glmnet package in R (version 4.3.1) with family = binomial for binary classification. To prevent data leakage and ensure unbiased feature selection, all LASSO analyses were performed within a 10-fold cross-validation (CV) framework (nfolds = 10). The optimal penalty parameter λ (lambda.min) was determined by minimizing the cross-validated binomial deviance across the 10 CV folds. An alternative penalty parameter λ.1se (the largest λ within one standard error of lambda.min) was also considered to evaluate the stability of feature selection under more stringent regularization. A fixed random seed (set.seed = 123) was used to ensure reproducibility.

The LASSO coefficient path (**Figure 4B**) illustrates the dynamic changes of each protein’s coefficient as a function of log(λ): with increasing penalty strength (decreasing log(λ)), the coefficient of TNFRSF11A was first shrunk to zero, while the coefficients of the remaining proteins gradually decreased. At the optimal lambda.min, six proteins (MERTK, BOC, TNFRSF10A, CCL3, AGRP, and PD-L2) retained non-zero coefficients, forming the final feature subset; TNFRSF11A was excluded due to its weaker predictive contribution or redundancy with other selected features. The feature selection was further validated using the 1-standard-error rule (lambda.1se), which yielded a subset of four proteins(MERTK, BOC, TNFRSF10A, and CCL3), confirming the robustness of the key predictors.

### 3.2 Age-related differentially expressed proteins in T2DM patients

Aging is an independent risk factor for CVD, and both the incidence of diabetes and the prevalence of its complications are significantly increased in the elderly population. In China, the prevalence of diabetes among elderly individuals is as high as 23.7% (21); indeed, approximately 45.6% of elderly diabetic patients remain undiagnosed (22). Moreover, elderly diabetic patients face a significantly elevated risk of cardiovascular disease, cerebrovascular disease, and microvascular complications (23).

Previous studies have demonstrated that the prevalence of T2DM increases markedly after the age of 55 years (24), and this age is widely recognized as a critical turning point for age-related changes in immune function and metabolic status. Indeed, 55 years is commonly adopted as the threshold for elderly status in numerous aging-related studies (25–27). Accordingly, in the present study, 66 patients with type 2 diabetes were stratified into two groups using a cutoff age of 55 years: <55 years (n = 38) and ≥55 years (n = 28), to investigate age-associated protein expression profiles.

#### 3.2.1 Baseline demographic and clinical characteristics

To investigate age-associated protein expression differences in type 2 diabetes patients, 66 patients were stratified into <55 years (n = 38) and ≥55 years (n = 28) groups. Baseline characteristics are presented in **Table 2**. The two groups were comparable with respect to BMI (P > 0.05), waist circumference (P > 0.05), hip circumference (P > 0.05), HbA1c (P > 0.05), and hypoglycemic medication use (P > 0.05), indicating that observed differences in protein expression are primarily attributable to age rather than metabolic or medication confounding. FPG differed significantly between groups (P < 0.05) and was therefore adjusted for in subsequent multivariable analyses. Diabetes duration was longer in the ≥55 years group, approaching statistical significance (P = 0.062).

**Table 2:** Baseline characteristics of study participants stratified by age group.

Variable	<55 years (n = 38)	≥55 years (n = 28)	P value
Age, years	43.95 ± 9.43	60.29 ± 3.38	**< 0.001**
Sex, male, n (%)	23(53.5%)	20(46.5%)	0.511
BMI, kg/m²	24.82 ± 3.65	25.19 ± 2.98	0.517
Waist circumference, cm	90.88 ± 9.75	91.68 ± 10.04	0.748
Hip circumference, cm	99.50 ± 8.58	98.54 ± 5.64	0.730
HbA1c, %	8.68 ± 2.56	9.45 ± 2.45	0.179
FPG, mmol/L	8.61 ± 3.05	10.57 ± 3.54	**< 0.05**
Diabetes duration, years	6.06 ± 3.90	9.43 ± 6.50	0.062
Any hypoglycemic agent, n (%)	31 (81.6%)	25 (89.3%)	0.606
Insulin, n (%)	9 (23.7%)	12 (42.9%)	0.166
Metformin, n (%)	19 (50.0%)	13 (46.4%)	0.970

Data are presented as mean ± SD or n (%). P values were calculated using Student's t-test or Mann-Whitney U test for continuous variables and Chi-square test for categorical variables. Bold values indicate statistical significance (P < 0.05).

#### 3.2.2 Differential protein screening

To identify age-associated protein candidates, we performed Wilcoxon rank-sum test comparing the <55 years (n = 38) and ≥55 years (n = 28) groups. A total of 14 proteins showed significant expression differences at P < 0.05. Functional annotation indicated that these proteins are primarily enriched in inflammatory responses (CCL3, IL-1ra, CCL4, CCL11), lipid metabolism (LPL), vascular stress (IL-8), and immune regulation (VSIG2, CD4). These findings suggest that age-related molecular changes in type 2 diabetes involve multiple interconnected pathways beyond glycemic control.

To exclude potential confounding effects, we constructed four stepwise-adjusted linear regression models to evaluate the association between age group (<55 vs ≥55 years) and the 14 preliminarily identified proteins. Model 1 was unadjusted; Model 2 adjusted for sex, BMI, and HbA1c; Model 3 further adjusted for medication use (yes/no); and Model 4 further adjusted for metformin and insulin use. In the fully adjusted model (Model 4), nine proteins remained significantly associated with age group at FDR < 0.05: VSIG2 (β = 0.432, P = 0.0013), LPL (β = 0.429, P = 0.0024), S100A11 (β = 0.770, P = 0.0028), CST5 (β = 0.313, P = 0.0017), TNFRSF21 (β = 1.346, P = 0.0151), IL-8 (β = 0.154, P = 0.0163), S100A5 (β = 0.192, P = 0.0211), S100A17 (β = -1.117, P = 0.0265), and S100Z (β = -0.456, P = 0.0317). Notably, VSIG2, LPL, S100A11, and CST5 exhibited FDR values below 0.01, indicating the most robust associations with age. These proteins are functionally involved in immune regulation (VSIG2), lipid metabolism (LPL), inflammatory responses (IL-8), and calcium signaling (S100 family), consistent with the age-related metabolic and inflammatory changes observed in type 2 diabetes (**Table 3**).

**Table 3:** Multivariable linear regression models for age-associated proteins.

Protein	Model 1	Model 2	Model 3	Model 4
VSIG2	β=+0.418, FDR = 0.009	β=+0.459, FDR = 0.006	β=+0.462, FDR = 0.005	β=+0.432, FDR = 0.010
LPL	β=+0.419, FDR = 0.009	β=+0.454, FDR = 0.006	β=+0.462, FDR = 0.005	β=+0.429, FDR = 0.010
S100A11	β=+0.667, FDR = 0.021	β=+0.768, FDR = 0.007	β=+0.777, FDR = 0.006	β=+0.770, FDR = 0.010
CST5	β=+0.295, FDR = 0.009	β=+0.301, FDR = 0.007	β=+0.306, FDR = 0.006	β=+0.313, FDR = 0.010
TNFRSF21	β = +1.270, FDR = 0.022	β = +1.239, FDR = 0.021	β = +1.254, FDR = 0.023	β = +1.346, FDR = 0.038
IL-8	β = +0.163, FDR = 0.022	β = +0.183, FDR = 0.017	β = +0.184, FDR = 0.017	β = +0.154, FDR = 0.038
S100A5	β = +0.172, FDR = 0.037	β = +0.199, FDR = 0.021	β = +0.198, FDR = 0.023	β = +0.192, FDR = 0.042
S100A17	β = -1.135, FDR = 0.025	β = -1.295, FDR = 0.020	β = -1.295, FDR = 0.021	β = -1.117, FDR = 0.046
S100Z	β = -0.430, FDR = 0.037	β = -0.502, FDR = 0.021	β = -0.501, FDR = 0.023	β = -0.456, FDR = 0.049

Model 1: Unadjusted. Model 2: Adjusted for sex, BMI, and HbA1c. Model 3: Further adjusted for medication use (yes/no). Model 4: Further adjusted for metformin and insulin use. β coefficients represent the change in protein expression (NPX) in the ≥55 years group relative to the <55 years group. FDR, false discovery rate (Benjamini-Hochberg adjusted).

#### 3.2.3 Construction and validation of the final predictive model

**Figure 5:**
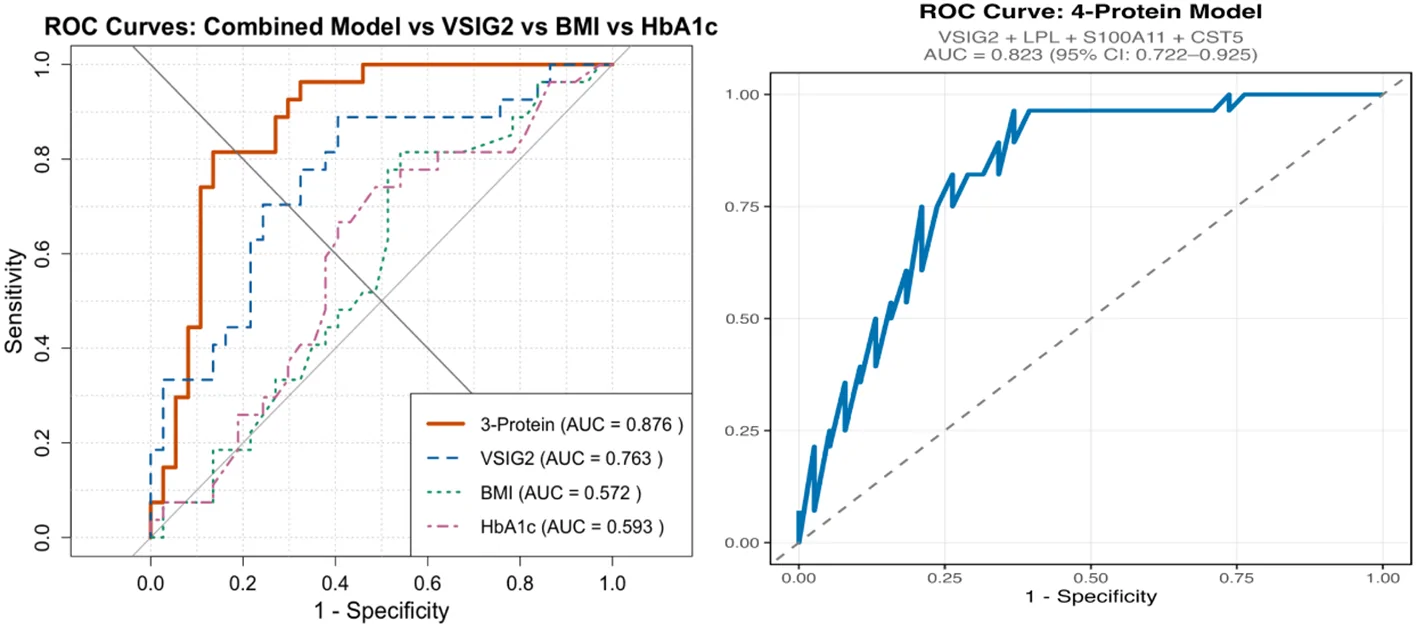
ROC curves comparing different panels and clinical markers. The 3-protein panel (VSIG2 + S100A11 + S100A5) showed the best performance (AUC = 0.876, 95% CI: 0.786-0.961), compared with the 4-protein panel (AUC = 0.823, 95% CI: 0.722-0.925), VSIG2 (AUC = 0.736), BMI (AUC = 0.572), and HbA1c (AUC = 0.593), demonstrating superior and more parsimonious age discrimination.

To construct a parsimonious prediction model, we first incorporated the four most robust age-associated proteins (VSIG2, LPL, S100A11, and CST5) into a logistic regression model. This 4-protein panel achieved an AUC of 0.823 (95% CI: 0.722–0.925), significantly outperforming individual proteins (VSIG2: AUC = 0.737; LPL: AUC = 0.731; S100A11: AUC = 0.712; CST5: AUC = 0.704). However, in this model, only S100A11 (P = 0.0328) and VSIG2 (P = 0.0557) showed notable contributions, whereas LPL and CST5 were no longer significant after adjustment, likely due to moderate correlation between these proteins (LPL vs CST5, r = 0.641). To further optimize the model, we replaced the redundant proteins with S100A5, which exhibited a negative association with age (β = −3.015, P = 0.005) and thus provided complementary information. The resulting 3-protein model (VSIG2 + S100A11 + S100A5) achieved an AUC of 0.874 (95% CI: 0.786–0.961), notably higher than that of the 4-protein model (**Figure 5**).

At the optimal cutoff determined by the Youden index, the 3-protein model achieved a sensitivity of 0.821, a specificity of 0.838, an accuracy of 0.831, and an F1 score of 0.807. Bootstrap internal validation with 1000 resamples confirmed good model stability (optimism = −0.023, corrected AUC = 0.899) and ruled out overfitting (overfitting index = −2.65%).

#### 3.2.4 Model validation

**Figure 6:**
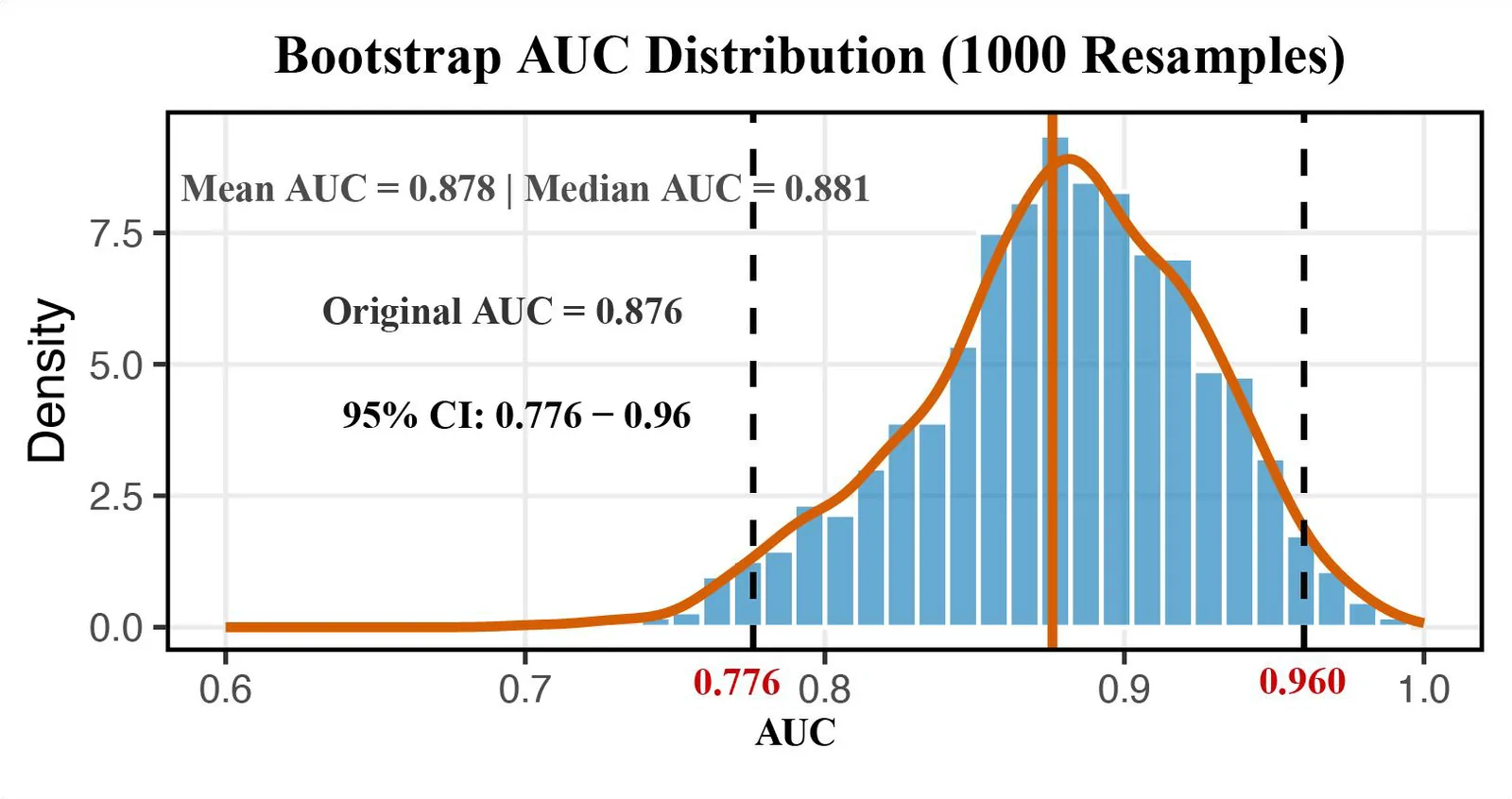
Bootstrap internal validation AUC distribution for the 3-protein model. The red solid line represents the original AUC (0.876), and the black dashed lines represent the Bootstrap 95% CI (0.776-0.960). The AUC distribution was approximately normal with a mean of 0.878 and a median of 0.881, indicating good model stability and generalizability without overfitting.

To assess model stability and overfitting risk, Bootstrap internal validation with 1000 resamples was performed. The original AUC of the 3-protein model was 0.876, with a Bootstrap-corrected AUC of 0.900. The optimism was-0.024, and the overfitting index was -2.65%, indicating negligible overfitting. The Bootstrap 95% confidence interval for the AUC was 0.776-0.960 (**Figure 6**).

## 4 Discussion

In this study, Olink proteomics was employed to compare serumprotein profiles between diabetic patients and healthy controls. Sixteen differentially expressed proteins were identified using stringent criteria (|log_2_FC| > 0.585, VIP > 1.0, FDR < 0.05),and six proteins (MERTK, BOC, TNFRSF10A, CCL3, AGRP, andPD-L2) were further selected as key contributors to diabetes diagnosis via random forest and LASSO regression.

Building on this, we focused on age-associated molecular changes within the diabetic population. Stratifying 66 T2DM patients into <55 years (n = 38) and ≥55 years (n = 28) groups, we performed age-specific protein screening and constructed a multi-protein model for age discrimination. Wilcoxon rank-sum test initially identified 14 age-associated differentially expressed proteins. After stepwise adjustment for sex, BMI, HbA1c, and hypoglycemic medications using four linear regression models, nine proteins remained independently associated with age group (FDR < 0.05), with VSIG2, LPL, S100A11, and CST5 showing the most robust associations (FDR < 0.01). The four-protein panel achieved an AUC of 0.823. After further optimization, the three-protein model (VSIG2 + S100A11 + S100A5) improved the AUC to 0.874 (95% CI: 0.786–0.961), substantially outperforming traditional markers BMI (AUC = 0.572) and HbA1c (AUC = 0.593). Bootstrap internal validation with 1000 resamples confirmed good model stability (optimism = −0.024, corrected AUC = 0.900, overfitting index = −2.65%), suggesting its potential clinical applicability.

VSIG2 (V-set and immunoglobulin domain-containing 2), a member of the immunoglobulin superfamily, is a newly identified immune checkpoint molecule. Acting as a ligand, it suppresses T cell activation and regulates the infiltration of B cells and M1-type macrophages, thereby exerting vital functions in age-related immunosenescence (28–30). Epidemiological evidence has indicated that elevated VSIG2 levels are strongly associated with incident cardiovascular disease and increased mortality risk (31). Notably, circulating VSIG2 correlates with the risk of major adverse cardiovascular events (MACE) among elderly patients with advanced kidney disease (32). In the present study, VSIG2 was significantly upregulated in patients with T2DM aged ≥55 years (β=0.432, FDR = 0.0099), and this association remained robust after adjustment for multiple confounding variables. These findings suggest that aging is accompanied by persistent remodeling of the immune system manifested as chronic low-grade inflammation and immune cell dysfunction. We therefore hypothesize that VSIG2 serves as a key regulatory molecule mediating age-associated immune remodeling.

Lipoprotein lipase (LPL) is a core effector enzyme in lipid metabolism that catalyzes the hydrolysis of triglyceride-rich lipoproteins in circulation, and its function is tightly regulated by ANGPTL family protein complexes (33, 34). Previous *in vitro* studies have demonstrated hypermethylation of the LPL gene in adipose-derived stem cells (ASCs) and dedifferentiated fat (DFAT) cells isolated from aged donors, indicating that aging represses LPL transcription via epigenetic modification and impairs lipid metabolism and adipogenic differentiation capacity of adipose stem cells (35). In the present study, LPL protein abundance was significantly elevated in participants aged ≥55 years (β=0.429, FDR = 0.0099), confirming that age-induced lipid metabolic remodeling is accompanied by altered LPL expression. Notably, accumulating evidence consistently shows that LPL enzymatic activity declines progressively with advancing age, whereas there remains no consensus regarding the pattern of LPL protein expression during senescence (36). It is speculated that upregulated LPL protein represents an adaptive regulatory mechanism that counteracts age-related lipid accumulation and compensates for the reduction in LPL activity. This observation also suggests that circulating LPL levels may serve as a potential biomarker for identifying age-associated lipid disorders. Integrating evidence from human proteomics and stem cell epigenetic investigations, LPL acts as a critical molecule linking aging, dysfunction of adipose stem cells, and disorders of glucose and lipid metabolism.

S100A11, S100A5, S100A17 and S100Z belong to the S100 calcium-binding protein family, which participates in calcium signal transduction, inflammatory regulation and cell differentiation (37). Among them, S100Z directly modulates the proliferation and apoptosis of pancreatic β-cells and contributes to the pathogenesis of type 2 diabetes mellitus (38). S100A11 activates multiple inflammatory signaling pathways, and its elevated expression serves as a molecular signature of inflammaging (39). S100A5 is predominantly expressed in the nervous system, and the biological implications of its age-dependent increase in serum levels remain to be further elucidated (40). In the present study, S100A11 (β=0.770, FDR = 0.0099) and S100A5 (β=0.192, FDR = 0.0421) were significantly upregulated in participants aged ≥55 years, whereas S100A17 (β=-1.117, FDR = 0.0464) and S100Z (β=-0.456, FDR = 0.0493) were markedly downregulated, indicating highly heterogeneous expression patterns of S100 family members during aging. Circulating S100Z levels change with age in patients with T2DM. Given that S100Z regulates the balance between pancreatic β-cell proliferation and apoptosis via calcium signaling, this molecule may act as a critical mediator linking aging, pancreatic islet dysfunction and the progression of T2DM. Meanwhile, multiple S100 family proteins exhibit age-related alterations simultaneously, suggesting that calcium signaling pathways mediated by the S100 family represent a common regulatory cascade governing diabetes-associated aging.

Cystatin 5 (CST5) is a member of the cysteine protease inhibitor family and participates in the maintenance of tissue homeostasis via regulating protease activity (41). Accumulating evidence has validated CST5 as an ultra-early inflammatory biomarker for traumatic brain injury (TBI) and persistent atrial fibrillation (PeAF) (42, 43). Additionally, CST5 represents the immune marker most strongly associated with age among patients with major depressive disorder (44). In the present study, CST5 was significantly upregulated in participants aged ≥55 years (β=0.313, FDR = 0.0099), implying that age-related protease–antiprotease homeostasis disturbance is accompanied by elevated CST5 expression. Mendelian randomization studies further demonstrate that increased CST5 levels causally reduce circulating brain-derived neurotrophic factor (BDNF), thereby triggering neurodegeneration and cognitive impairment (45). Collectively, the age-dependent upregulation of CST5 not only reflects the remodeling of the protease system during senescence but may also promote inflammaging and metabolic-related target organ injury by suppressing BDNF-mediated neuroprotective pathways.

Based on the aforementioned differentially expressed proteins, a three-protein panel consisting of VSIG2, S100A11 and S100A5 was established for age stratification in patients with T2DM. The combined panel exhibited favorable discriminative performance (AUC = 0.874), which was significantly superior to conventional clinical indicators including BMI and HbA1c. This model only requires quantification of three plasma proteins, conferring advantages such as minimal invasiveness, convenient operation and favorable repeatability, and thus possesses promising translational potential. Compared with the four-protein model (VSIG2 + LPL + S100A11 + CST5), the three-protein model achieved an AUC increase of 0.051 with fewer detected markers, indicating that inclusion of excessive biomarkers may lead to information redundancy and impair predictive performance. S100A5 was negatively correlated with age (β = −3.015), which complemented the upregulated proteins and provided distinct biological information, potentially accounting for the improved predictive ability of this parsimonious model. This efficient biomarker panel may facilitate the evaluation of biological age and identification of high-risk individuals among T2DM patients, supporting the delivery of individualized management strategies.

Internal validation with 1000-resample bootstrap confirmed favorable stability and generalizability of the three-protein model. The optimism value was −0.024, suggesting that the average performance in resampled datasets was no worse than that in the original cohort without obvious overfitting. The bias-corrected AUC reached 0.900, exceeding the original AUC (0.874) and further verifying model robustness. The overfitting index was −2.65%, far below the critical threshold of 5%, which further supported its generalizability. In terms of calibration, the Hosmer–Lemeshow test yielded P = 0.024, slightly less than 0.05, indicating mild miscalibration, which may be attributable to the limited sample size and insufficient observations within low-probability intervals. Calibration curves demonstrated satisfactory fitting across the predicted probability range of 0.3–0.8, suggesting acceptable overall clinical applicability. The discriminative ability of the three-protein model (AUC = 0.874) markedly outperformed BMI (0.572) and HbA1c (0.593), highlighting the limitations of conventional metabolic indicators in capturing age-related biological alterations. BMI serves only as a crude measure of adiposity and is susceptible to multiple confounders, making it difficult to precisely reflect age-mediated metabolic remodeling; HbA1c primarily reflects recent glycemic control and exhibits weak correlation with biological age. In contrast, proteomic biomarkers directly capture perturbations in aging-associated molecular pathways and provide in-depth biological insights that cannot be achieved by routine clinical parameters.

## 5 Study advantages and limitations

The present study has multiple strengths. First, protein detection was performed using the Olink targeted proteomics platform, which offers high sensitivity and specificity and ensures the reliability of quantitative protein data. Second, rigorous statistical strategies were applied by constructing four gradually adjusted models with progressive incorporation of confounding variables, which minimized confounding bias and guaranteed the robustness of the association results. Third, the development and optimization of the multi-protein predictive model followed a systematic and standardized workflow. The candidate markers were stepwise filtered from 14 age-related proteins to four core molecules, and finally optimized into a parsimonious three-protein signature, achieving a clear and reproducible screening procedure. Fourth, sufficient internal validation was conducted using a 1000-resample bootstrap method to evaluate model stability and generalizability, further verifying the credible discriminative performance of the signature. Fifth, all identified candidate proteins have well-annotated biological functions, and their involved pathways are closely consistent with the mechanisms underlying aging and type 2 diabetes mellitus, supporting the biological rationality of our findings.

Nevertheless, several limitations of this study should be acknowledged. First, the relatively limited sample size may restrict statistical power and model stability to a certain extent. Although bootstrap internal validation demonstrated favorable model robustness, external validation based on larger independent cohorts is still required. Second, this cross-sectional observational study cannot clarify the temporal causality between aging and altered protein expression, and bidirectional interactions between age and molecular changes may exist. Third, only 92 target proteins were detected in the current panel, which may leave other key molecules associated with age-related metabolic disorders unrecognized; future untargeted proteomic profiling is warranted to explore additional potential biomarkers. Fourth, external cohort validation was not performed in the present study, and the extrapolation ability of the model remains to be further validated in populations from different regions and ethnicities. Fifth, all participants enrolled in this study were diagnosed with type 2 diabetes mellitus; therefore, the current findings cannot be directly generalized to patients with type 1 diabetes or healthy individuals. Further studies with expanded population subtypes are needed for comparative verification.

## 6 Future perspectives

The findings of the present study can be further extended and deepened in multiple dimensions. First, multicenter and large-scale prospective cohorts are needed to externally validate the clinical applicability and population generalizability of the three-protein signature. Longitudinal observational designs will help dynamically characterize age-dependent protein expression patterns and clarify their temporal correlations with the occurrence and progression of diabetic complications. Second, in-depth functional experiments, including gene knockout and overexpression assays, are required to validate the causal mechanisms by which core proteins (VSIG2, S100A11, S100A5, and LPL) mediate aging and T2DM progression, and to explore their potential as novel therapeutic targets for diabetic aging. Third, integrating the established proteomic signature with conventional clinical risk scoring systems may generate a more comprehensive and precise predictive model, which can improve biological age stratification and facilitate individualized clinical management for patients with T2DM.

## 7 Conclusions

In conclusion, the present study systematically profiled age-related plasma protein alterations in patients with T2DM using Olink targeted proteomics. Nine proteins, including VSIG2, LPL, S100A11, CST5, TNFRSF21, IL-8, S100A5, S100A17, and S100Z, were independently associated with age grouping (FDR < 0.05), among which VSIG2, LPL, S100A11, and CST5 exhibited the most robust associations (FDR < 0.01). A parsimonious three-protein signature comprising VSIG2, S100A11, and S100A5 was constructed and showed excellent discriminative efficiency for age stratification (AUC = 0.874), which was significantly superior to conventional clinical indicators including BMI and HbA1c. Bootstrap internal validation confirmed the favorable stability and generalizability of the signature. This minimally invasive and cost-effective three-protein detection model holds great potential for clinical application to evaluate biological aging status in T2DM patients. Functionally, these dysregulated proteins are critically involved in immune modulation (VSIG2), lipid metabolism (LPL), inflammatory response (IL-8), and calcium signaling transduction (S100 family). Collectively, the present findings provide novel mechanistic insights into the molecular basis of diabetes-associated aging.

## Data Availability

The original contributions presented in the study are included in the article/**Supplementary Material**. Further inquiries can be directed to the corresponding authors.
