# Supplementary material for: Olink proteomics identifies serum protein biomarkers and an age-stratified diagnostic model for diabetes in Chinese patients
Source: Front Endocrinol (Lausanne). 2026 Aug 26;17:1900153. doi: 10.3389/fendo.2026.1900153 (PMC13553404; doi:10.3389/fendo.2026.1900153)
Supplement: Supplementary file 1 [file Table1.docx]

**Supplementary table S1**

### **Table S1Internal validation results of the 3‑protein model**

| **Validation Method** | **Mean AUC ± SD** | **Median AUC (IQR)** | **95% CI** | **Key Finding** |
| --- | --- | --- | --- | --- |
| Bootstrap (1000 resamples) | 0.878 ± 0.047 | — | 0.776–0.960 | Optimism = −0.024, corrected AUC = 0.900 |
| 10‑fold CV (100 repeats) | 0.857 ± 0.151 | 0.903 (0.750–1.000) | 0.500–1.000 | Median AUC consistent with Bootstra |

CV: cross‑validation; SD: standard deviation; IQR: interquartile range; CI: confidence interval

To evaluate the generalizability of the 3‑protein model, repeated 10‑fold cross‑validation (100 repetitions) was performed. The model achieved a median AUC of 0.903 (IQR: 0.750–1.000), which was highly consistent with the Bootstrap‑corrected AUC (0.900). Further analysis revealed that 75.0% of the cross‑validation models achieved AUC ≥ 0.75, and 50.0% achieved AUC ≥ 0.90, indicating robust discriminatory ability across most data partitions. Although the mean AUC was 0.857 ± 0.151, reflecting the variability expected with a modest sample size (n = 66), the median AUC (0.903) provides a more robust estimate of central tendency, less influenced by extreme values observed in small data partitions. Taken together, the Bootstrap and cross‑validation results consistently confirm the favorable stability and generalizability of the 3‑protein model.
